# Prevalence of group B streptococcus colonization in pregnant women in Jiangsu, East China

**DOI:** 10.1186/s12879-021-06186-5

**Published:** 2021-05-27

**Authors:** Yanmei Ge, Fei Pan, Rui Bai, Yuan Mao, Wenli Ji, Fenfang Wang, Huacheng Tong

**Affiliations:** 1 Nanjing Tongren Hospital, School of Medicine, Southeast University, Nanjing, 211102 China; 2 Nanjing Kingmed Center for Clinical Laboratory Co., Ltd, Nanjing, 210043 China

**Keywords:** Group B streptococcus, Prevalence, Colonization, Antibiotic resistance

## Abstract

**Background:**

Group B streptococcus (GBS) is the leading cause of early-onset neonatal sepsis. However, GBS was infrequently reported in the developing world in contrast to western countries. This study assessed the prevalence of GBS colonization among pregnant women in Jiangsu, East China, and revealed the difference of GBS infection between culture and PCR.

**Methods:**

A total of 16,184 pregnant women at 34 to 37 weeks’ gestation aged 16–47 years were recruited from Nanjing Kingmed Center for Clinical Laboratory. Nine thousand twenty-two pregnant women received GBS screening by PCR detection only. Seven thousand one hundred sixty-two pregnant women received GBS screening by bacterial culture and GBS-positive samples were tested for antibiotic resistance.

**Results:**

The overall GBS positive rate was 8.7% by PCR and 3.5% by culture. Colonization rate was highest in the “25–29 years” age group. The 249 GBS-positive samples which detected by culture were all sensitive to penicillin. The prevalence of resistance to erythromycin, clindamycin, and levofloxacin was 77.5, 68.3, and 52.2%, respectively.

**Conclusions:**

This study revealed the data on the prevalence of GBS colonization in pregnant women at 34 to 37 weeks’ gestation in Jiangsu, East China. It compared the difference of the sensitivity to detect GBS between PCR and culture. PCR was expected to become a quick method in pregnancy women conventional detection of GBS infection.

## Background

Group B streptococcus (GBS) is the main pathogen of perinatal infection. It is not only the leading cause of early-onset neonatal sepsis and meningitis (first 28 days of life), but also has been associated with preterm labor, premature rupture of membranes, chorioamnionitis, and puerperal and fetal infections in many countries [1–3]. Screening of pregnant women for GBS colonization during the third trimester, coupled with targeted intrapartum antibiotic prophylaxis (IAP) of colonized women during labor, has reduced the incidence of invasive GBS disease in western countries [4]. GBS detection and identification has become more commonplace, due to the availability of polymerase chain reaction (PCR) technology [5]. However, the traditional method of culture of GBS is still the gold standard.

Penicillin and clindamycin are the first line of antibiotic recommendations in most countries. Penicillin, ampicillin, and cefepime are the main drugs of choice to treat GBS infection in China. Vancomycin, macrolides (such as erythromycin, azithromycin, and clarithromycin), and lincosamides (clindamycin) may be used as the alternative drugs for patients allergic to penicillin or cephalosporins [6–9].

In this study, we investigated the GBS colonization rate in pregnant women in Jiangsu, China. At the same time, we compared the difference in the detection rate of GBS between the two methods of culture and PCR and described the sensitivity of GBS to different antibiotics.

## Methods

### Study population

Our study is retrospective study. Between June of 2017 and June of 2019, pregnant women at 34 to 37 weeks’ gestation who resided in Jiangsu Province and received GBS screening at Nanjing Kingmed Center for Clinical Laboratory were studied. The pregnant women had not received antibiotic treatment for at least 2 weeks before recruitment into the study [10]. We performed an analysis of 16,184 women aged 16–47 years, including 9022 pregnant women who received GBS screening by PCR and 7162 by culture. GBS-positive samples were tested for antibiotic resistance by automatic microbial identification and drug sensitivity analysis system.

### Specimen collection

A set of vagino-rectal swab samples consisting of two swabs were taken. The specific operation steps were carried out according to the method recommended by the 2002 CDC. The accurately labeled swabs were placed in a cooler box containing ice packs, and transported to the laboratory at Nanjing Kingmed Center within 2–4 h of collection. Specimens were collected by an obstetrician and taken as part as standard care, for 2 years from 16,184 pregnant women.

### PCR assays

GBS DNA was detected using the Group B Streptococcus (GBS) nucleic acid detection kit (BioChain (Beijing) Science & Technology. Inc.). Firstly, each vaginal or rectal swab specimen was combined with 1 ml of normal saline (0.9% NaCl). 500 μl of the vaginal specimen was mixed with 500 μl of rectal swab specimen. DNA was extracted from the mixed liquid following the manufacturer’s instructions, then 100 ng (5 μl) GBS DNA was used as a template and added into 35 μl reaction mixture**.** Primer and probe referred to the previous study [11]. The conditions for the PCR were as follows: 50 °C for 2 min, initial denaturation 95 °C for 5 min, 45 cycles of PCR at 95 °C for 15 s and 60 °C for 35 s. Positive reactions were defined as a cycle threshold (CT) < 38. Negative control reactions (no DNA template) were included with every run. PCR was done on an ABI PCR system 7500 version 2.3 for the amplification.

### Microbiology (culture)

Cotton swab samples (a set of vagino-rectal swab samples) from pregnant mothers were inoculated into Todd-Hewitt culture broth, subcultured on Columbia blood agar to which 5% sheep blood has been added (Oxoid, United Kingdom), then incubated at 37 °C in ambient air for 24-48 h. The colonies on the solid media were presumptively identified as Group B Streptococcus if they forming light red to dark red colonies on CHROMagarStrepB.

### Antimicrobial susceptibility test

GBS-positive samples were tested for antibiotic resistance by VITEK 2 Compact system (France). The disk diffusion method was used to measure resistance to penicillin, ampicillin, cefepime, cefotaxime, ergomycin, clindamycin, chloramphenicol, linezolid, vancomycin, and levofloxacin according to the Clinical and Laboratory Standards Institute (CLSI) standards [12].

### Statistical analysis

Statistical analyses were performed using SPSS version 19.0 (IBM, Armonk, NY, USA). GBS positive rate was estimated by a proportion and summarized as a percentage and proportions compared using exact binomial 95% confidence intervals (95% CI). The chi-squared (*x*^*2*^) was used to compare the proportions of different age groups. A *p*-value of < 0.05 was considered statistically significant.

## Results

### The prevalence of GBS infection

A total of 16,184 pregnant women were enrolled in the study. Seven hundred eighty-nine participants (8.7, 95% CI: 8.2–9.3%) out of 9022 women studied by PCR showed GBS colonization, while 249 (3.5, 95% CI: 3.1–3.9%) of 7162 women investigated by the culture were colonized (Table 1). The average positive rate of GBS infection is 6.4% (95% CI: 6.0–6.8%).

**Table 1:** The prevalence of GBS infection in all the specimens

Participants	Total	Positive	% (95%CI)
PCR	9022	789	8.7 (8.2–9.3)
Culture	7162	249	3.5 (3.1–3.9)

### Prevalence of GBS colonization among pregnant women of different age groups

The analysis of the prevalence of positive GBS results was presented by different age groups (≤24 years, 25–29 years, 30–34 years, 35–39 years, and ≥ 40 years). There were both no obvious difference among different age groups by PCR (*P* = 0.161) and by culture (*P* = 0.28).

The highest prevalence was found in the “25–29 years” age group (9.4, 95% CI: 8.5–10.4%), while the lowest prevalence was found in the “younger than 24 years” age group (7.5, 95% CI: 6.3–8.6%) based on the PCR method. However, based on the culture method, the prevalence rates were 3.6 and 3.2%, respectively. However, the group with the highest frequency was found in the “older than 40 years” age group (7.1, 95% CI: 2.0–12.3%), based on the culture method. This group (≥40 years) showed a smaller difference in the prevalence rates between the culture method and PCR method (1.6%), meanwhile, this difference was greater than 4% in the other groups (Table 2).

**Table 2:** Prevalence of GBS colonization in different age groups

Age Groups (Years)	PCR		Culture	
	n (Postive/Total)	Prevalence Rate (95% CI)	n (Postive/Total)	Prevalence Rate (95% CI)
≤24	148/1982	7.5 (6.3–8.6)	54/1710	3.2 (2.3–4.0)
25–29	377/3991	9.4 (8.5–10.4)	124/3398	3.6 (3.0–4.3)
30–34	189/2193	8.6 (7.4–9.8)	49/1527	3.2 (2.3–4.1)
35–39	64/730	8.8 (6.7–10.8)	15/429	3.5 (1.8–5.2)
≥40	11/126	8.7 (3.7–13.7)	7/98	7.1 (2.0–12.3)
*P*	0.161		0.28	

*CI* confidence interval

Compare the age groups with the highest and lowest prevalence rate by PCR, *P* = 0.011

Compare the age groups with the highest and lowest prevalence rate by Culture, *P* = 0.034

### Antimicrobial susceptibility

Antimicrobial susceptibility testing identified all samples detected by microbiology as susceptible to penicillin, linezolid, and vancomycin. The prevalence of resistance to erythromycin, clindamycin, and levofloxacin was 77.5, 68.3, and 52.2%, respectively (Table 3).

**Table 3:** The sensitivity of GBS to different antibiotic

Antibiotic	Total	S(%)	I(%)	R(%)
Penicillin	249	249 (100.0)	0 (0.0)	0 (0.0)
Ampicillin	246	243 (98.8)	0 (0.0)	3 (1.2)
Cefepime	250	247 (98.8)	0 (0.0)	3 (1.2)
Cefotaxime	235	233 (99.1)	0 (0.0)	2 (0.9)
Erythromycin	249	50 (20.1)	6 (2.4)	193 (77.5)
Clindamycin	249	76 (30.5)	3 (1.2)	170 (68.3)
Chloramphenicol	250	226 (90.4)	7 (2.8)	17 (6.8)
Linezolid	249	249 (100.0)	0 (0.0)	0 (0.0)
Vancomycin	244	244 (100.0)	0 (0.0)	0 (0.0)
Levofloxacin	224	103 (46.0)	4 (1.8)	117 (52.2)

*S* susceptible, *I* intermediate, *R* resistance

## Discussion

This study showed a low prevalence of GBS colonization in pregnant women in Jiangsu, East China. Colonization rate was highest among 25–29 years old by PCR, and culture. Our study also identified higher rates by PCR than by culture. PCR may, therefore, be expected to become a quick method to detect those at risk of GBS colonization in pregnancy compared to conventional detection of GBS infection. The GBS-positive samples which detected by culture were all sensitive to penicillin.

GBS infection can be transient or persistent during pregnancy, which inevitably leads to different results of GBS in the same pregnant woman at different times of pregnancy [1, 13]. Therefore, we should choose the same stage of pregnant women when studying the infection rate of GBS. There are regional differences in GBS colonization in pregnant women. For example, the reported prevalence of GBS for Africa is 22.4%, Southeast Asia is 11.1% and Taiwan is 23.7% [14, 15]. Unfortunately, large-scale multicenter epidemiological studies on maternal GBS colonization in mainland China are still rare [5].

So far, there have been many regional studies on the rate of GBS colonization in China. It was reported that the prevalence of GBS for Beijing was 7.1% and Qingdao in Shandong Province was 10.61% in Northern China [16, 17]; Shanghai was 3.7% and Nanjing was 4.16% in Eastern China [18, 19]; Chongqing was 7.05% and Chengdu in Sichuan Province was 5.02% in Southern China [20, 21]. The infection rates of GBS vary widely in different parts of China, and the prevalence of GBS in the northern region is significantly higher than in the eastern region. In our study, the rate of GBS colonization obtained by culture was 3.5% and that by PCR was 8.7%, in Jiangsu, China. The average positive rate of GBS infection was 6.4%. The rate in our study was lower than the northern region. The main reason for this difference may be related to local economic levels and environmental factors. Another important factor is the neglect of the detection method of GBS.

In our study, the rate of GBS colonization obtained by culture only (3.5%) was much lower than the rate obtained by PCR (8.7%) in Jiangsu, China. This is mainly because PCR is a rapid method which more sensitive and specific than culture. It may be due to the presence of nonviable GBS or low bacterial load in vaginal swabs, which cannot be detected by culture, but their DNA could be present for PCR amplification [22, 23]. Some pregnant women colonized by GBS might be missed only using a culture method.

Among the different age groups, the “25–29 years” age group had the highest colonization rate and should pay more attention. It may be related to the sexually active life, history of induced abortion, and higher estrogen levels during pregnancy in these age groups. These factors can cause micro-environmental changes in the genital tract bacteria. This phenomenon will continue to focus on future research. The “≥40 years” age group showed a smaller difference in the prevalence rates between the culture method and PCR method (1.6%), meanwhile, this difference was greater than 4% in the other groups. This may be due to the fact that this group (≥40 years) included a fewer cases for statistical analysis. So, the results of this group (≥40 years) were not suitable for comparative analysis.

IAP agents and dosing should be administered based on the test results of GBS among pregnant women according to the Centers for Disease Control (CDC) guidelines. Penicillin remains the agent of choice for IAP, with ampicillin as an acceptable alternative in China. Antimicrobial susceptibility testing should be ordered for antenatal GBS cultures performed on penicillin-allergic women at high risk for anaphylaxis. Then, the sensitive antibiotic could be chosen according to the results of antimicrobial susceptibility testing.

Previous studies on GBS bacteremia in adults from 2002 to 2010 in the USA had shown that erythromycin and clindamycin resistance occurred in 43.6 and 39.7% of cases, respectively [24]. And the prevalence of resistance to erythromycin and clindamycin from Taiwan for the period 2006–2008 was 58.3 and 57.9%, respectively [25]. In our study, the prevalence of resistance to erythromycin and clindamycin was 77.5 and 68.3%, respectively. It was higher than the prior studies. The goal of our research is pregnant women at 34 to 37 weeks’ gestation, which is a special group of people. It may be the main cause of this difference.

## Conclusion

In the present study, we presented the data on the prevalence of GBS colonization in pregnant women at 34 to 37 weeks’ gestation in Jiangsu, East China. At the same time, we compared the difference of GBS colonization between culture and PCR. Such data could guide interventions to control the prevalence of GBS. IAP agents and dosing should be administered according to the test results of GBS among pregnant women. As expected from the literature PCR has a higher sensitivity than culture, but does not allow assessment of antibiotic sensitivity. The very low prevalence of penicillin resistance suggests that PCR might be a very efficient screening test. Culture could be reserved to those pregnant women with allergy to penicillin.

## Data Availability

The data and materials used during the study are available from the corresponding author on reasonable request.

## Abbreviation

GBS: Group B Streptococcus
IAP: Intrapartum antibiotic prophylaxis
PCR: Polymerase chain reaction
CI: Confidence interval
